# Liver resection versus transarterial chemoembolization for huge hepatocellular carcinoma: a propensity score matched analysis

**DOI:** 10.1038/s41598-021-83868-9

**Published:** 2021-02-24

**Authors:** A. Bogdanovic, P. Bulajic, D. Masulovic, N. Bidzic, M. Zivanovic, D. Galun

**Affiliations:** 1 Clinic for Digestive Surgery, Clinical Center of Serbia, Koste Todorovica 6, Belgrade, 11000 Serbia; 2 Center for Radiology and Magnetic Resonance Imaging, Clinical Center of Serbia, Belgrade, 11000 Serbia; 3 School of Medicine, University of Belgrade, Belgrade, 11000 Serbia

**Keywords:** Cancer, Oncology

## Abstract

To date, it is unclear which treatment modality, liver resection (LR) or transarterial chemoembolization (TACE) is the more appropriate for patients with huge (≥ 10 cm) hepatocellular carcinoma (HCC). The study aim was to compare, using propensity score matching, short- and long-term outcomes of patients with huge HCC who underwent potentially curative LR or TACE. Patients with huge HCC who had been managed at the Clinical Center by curative-intent LR or by palliative TACE between November 2001 and December 2018 were retrospectively identified. The morbidity and mortality rates and overall survival were compared between the groups before and after the propensity score matching. Independent predictors of long-term survival were determined by multivariate analysis. A total of 103 patients with huge HCC were included; 68 were assigned to the LR group and 35 to the TACE group. The overall morbidity rate was higher in the LR group than in the TACE group before matching (64.7% vs. 37.1%, *p* = 0.012), while there was no difference after matching (60% vs. 30%, *p* = 0.055). The major morbidity and 30-days mortality were similar between the groups before and after matching. The LR group was associated with longer overall survival than the TACE group before matching (*p* = 0.032) and after matching (*p* = 0.023). Total bilirubin and TACE treatment were independent prognostic factors associated with long-term survival. In patients with huge HCC, liver resection provides better long-term survival than TACE and should be considered as the initial treatment whenever possible.

## Introduction

Hepatocellular carcinoma (HCC) is the sixth most common cancer and the third biggest cause of cancer-related death worldwide^1^. It is estimated that the number of new cases will increase from 841,080 diagnosed in 2018 to more than 1,300,000 cases in 2040^2^. Despite improvement in surveillance and diagnosis, the management of HCC continues to pose major problems for healthcare professionals^3^.

The modified Barcelona Clinic Liver Cancer (BCLC) staging system, endorsed by the European Association for the Study of the Liver, is one of the most widely utilized staging systems^4^. Once the diagnosis is established, the most appropriate treatment modality is allocated according to tumor burden, functional capacity of the liver and the patient general condition^5^. Liver resection, transplantation and thermal ablation, e.g. curative-intent treatment modalities, are feasible in a small percentage of HCC patients. However, the prognosis remains poor with an overall 5-year survival rate of approximately 5–6%^6^.

Approximately 90% of HCC cases are associated with chronic liver disease^7^. The comprehensive national screening programs intended for high-risk populations are implemented in developed countries, resulting in the increased number of HCC patients diagnosed at very early (BCLC 0) or early (BCLC A) stage. Thus, liver resection as a potentially curative treatment option can be offered to more HCC patients. In developing countries surveillance programs are lacking and HCC often develops in patients without known liver disease, resulting in late diagnosis of HCC. Patients are often diagnosed at symptomatic, advanced disease stage with large-size tumors^8^. According to the literature huge HCC is a tumor ≥ 10 cm in diameter^9^.

Tumors larger than 5 cm are beyond Milan criteria^10^ and are subsequently classified as intermediate BCLC stage. These patients are not good candidates for liver transplantation^11^. Moreover, patients with huge HCC are poor candidates for thermal ablation because it is difficult to achieve complete tumor necrosis of a large tumor nodule^12^. However, patients with a solitary HCC larger than 5 cm and preserved liver function, classified as intermediate stage, may benefit from liver resection^13^. Even in patients with portal vein tumor thrombus (type I and II) liver resection provides a survival benefit compared to TACE as it is indicated in a study performed on a population of 603 patients (1:2 ratio)^14^.

Currently, liver resection and transarterial chemoembolization (TACE) are the treatment modalities considered for patients with huge HCC. However, there is still debate about which is more appropriate. LR, although technically demanding, is a curative-intent modality that may improve overall survival^15^. Usually, more extended hepatectomy is required and often it is associated with higher rates of postoperative morbidity and mortality^16^. Unlike LR, TACE may result with limited therapeutic effect in patients with huge HCC because of the presence of extrahepatic collaterals. Therefore, in these cases it is difficult to achieve complete tumor embolization^17^.

The majority of published studies about the treatment of huge HCC have focused on outcomes after either LR or TACE^18–20^. There are few studies that have compared LR and TACE in the management of huge HCC^21,22^. These reports are mostly from the Asia–Pacific region, which favors LR. Results from Western countries, especially from middle-income countries characterized by a high percentage of hepatitis C-related HCC, are lacking.

The aim of this study was to compare short- and long-term outcomes of patients with huge HCC who underwent potentially curative LR or TACE. The comparison was done before and after propensity score matching to avoid selection bias.

## Results

**Figure 1:**
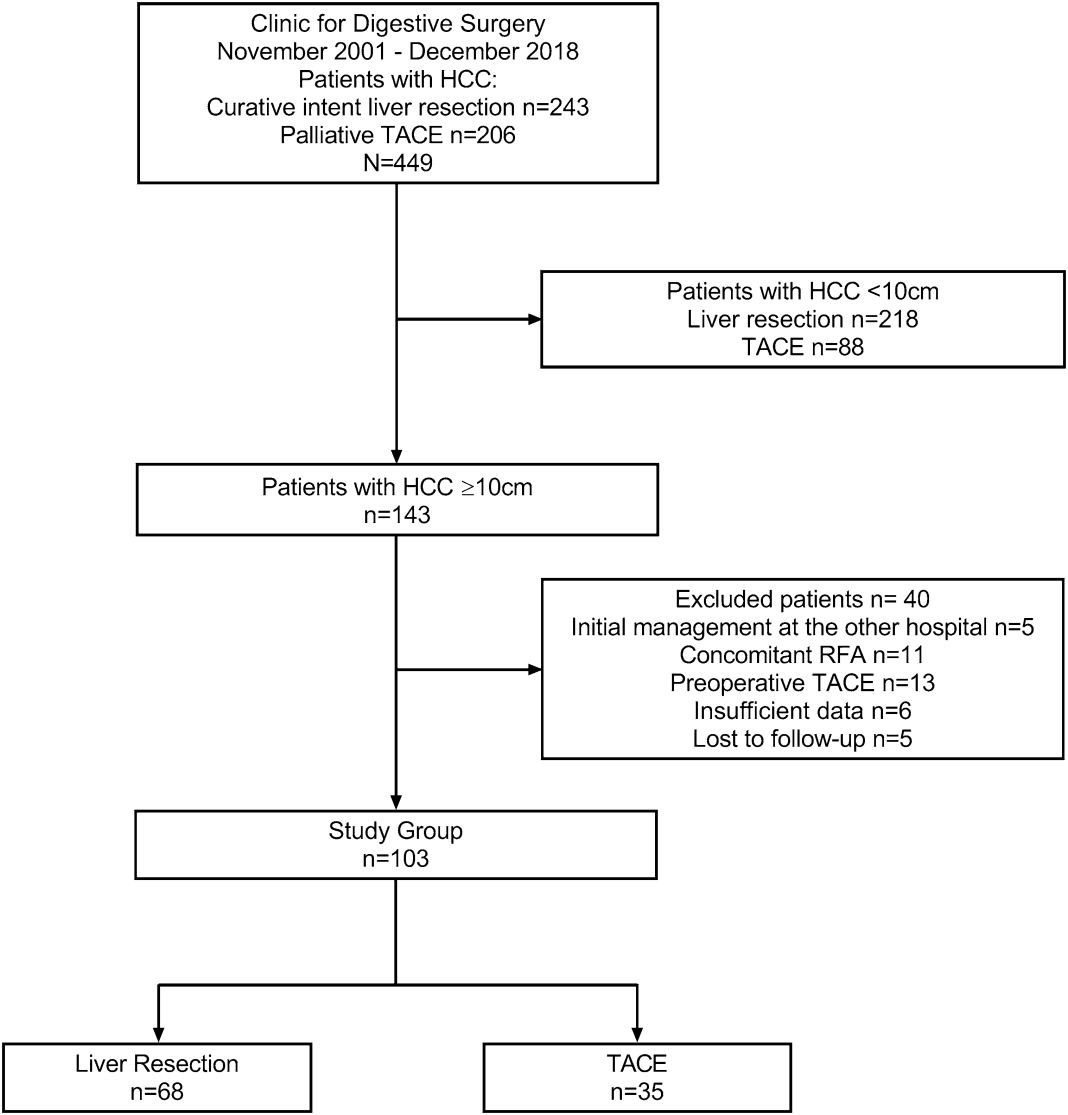
Study flow diagram. *HCC* hepatocellular carcinoma, *TACE* transarterial chemoembolization, *RFA* radiofrequency ablation.

One hundred forty three patients with huge HCC were included in the study. However, 40 patients were excluded because: 5 underwent initial management at the other hospital; 11 obtained concomitant radiofrequency ablation; 13 had preoperative TACE followed by curative-intent liver resection; 6 had insufficient data; and 5 were lost to follow-up. Among the remaining 103 patients, 68 underwent liver resection and were assigned to the LR group, and 35 underwent TACE and were assigned to the TACE group. Patient flow chart is shown in Fig. 1.

Demographic and clinical data are shown in detail in Table 1. Multiple lesions and bilobar distribution were more common in the TACE group (*p* = 0.001 and *p* ˂ 0.001, respectively). Serum γGT was higher in the TACE group (*p* = 0.003). Early BCLC stage was more common in the LR group while intermediate and advanced stage were more common in TACE group (*p* = 0.001). The two groups were similar with regard to age, sex, cirrhosis, hepatitis viral status, Child–Pugh score, ECOG status, major lesion size, albumin, total bilirubin, ALT and PT. The standardized mean difference was ˃ 20% for lesion number, distribution, major lesion size, ALT and γGT. Two groups were not well matched.

**Table 1:** Demographic and clinical features before propensity score matching.

	Total n = 103	LR group n = 68	TACE group n = 35	Standardized mean difference (%)	*p* value
*Age (year)*	64 (18–84)	64 (18–81)	65 (29–84)	19	0.471
*Sex, n (%)*					
Male	66 (64.1)	44 (64.7)	22 (62.9)	4	1
Female	37 (35.9)	24 (35.3)	13 (37.1)		
*Cirrhosis, n (%)*					
Yes	57 (55.3)	36 (52.9)	21 (60)	14	0.536
No	46 (44.7)	32 (47.1)	14 (40)		
*Viral status, n (%)*					
HCV	37 (35.9)	21 (30.9)	16 (45.7)	6	0.113
HBV	39 (37.3)	25 (36.8)	14 (40)		
Negative	27 (26.2)	22 (32.4)	5 (14.3)		
*Child–Pugh score, n (%)*					
A	100 (97.1)	67 (98.5)	33 (94.3)	18	0.266
B	3 (2.9)	1 (1.5)	2 (5.7)		
*ECOG status, n (%)*					
0	99 (96.1)	66 (97.1)	33 (94.3)	12	0.603
1	4 (3.9)	2 (2.9)	2 (5.7)		
AFP (ng/mL)	228 (2–40,000)	108.5 (2–31,000)	513 (3–40,000)	14	0.445
*Lesion number, n (%)*					
Solitary	88 (85.4)	64 (94.1)	24 (68.6)	54	0.001
Multiple	15 (14.6)	4 (5.9)	11 (31.4)		
*Distribution, n (%)*					
Unilobar	92 (89.3)	68 (100)	24 (68.6)	67	< 0.001
Bilobar	11 (10.7)	0	11 (31.4)		
*Major lesion size (cm), n (%)*					
10–14.9	70 (68)	48 (70.6)	22 (62.9)	32	0.139
15–19.9	22 (21.4)	11 (16.2)	11 (31.4)		
≥ 20	11 (10.7)	9 (13.2)	2 (5.7)		
*Albumin (g/L)*	39 (26–52)	39 (26–52)	38 (26–47)	7	0.772
*Total bilirubin (mmol/L)*	14 (5–236)	13 (6–236)	14 (5–78)	4	0.598
*ALT (IU/L)*	39 (6–426)	37 (6–228)	53 (6–426)	26	0.206
*γGT (IU/L)*	95 (9–2084)	62.5 (9–749)	138 (29–2084)	35	0.003
*PT*	12 (10–25)	12 (10–25)	12 (10–18)	4	0.710
*BCLC staging, n (%)*					
Early stage A	47 (45.6)	35 (97.2)	12 (57.1)		0.001
Intermediate stage B	6 (5.8)	1 (2.8)	5 (23.8)		
Advanced stage C	4 (3.9)	0	4 (19)		

Values are expressed as median (range) unless indicated otherwise.

*LR* liver resection, *TACE* transarterial chemoembolization, *HCV* hepatitis C virus, *HBV* hepatitis B virus, *ECOG* Eastern Cooperative Oncology Group, *AFP* alfa-feto protein, *ALT* alanine transaminase, *γGT* γ glutamil transferase, *PT* prothrombin time, *BCLC* Barcelona Clinic Liver Cancer.

Twenty pairs were identified using propensity score matching. Among those, the standardized mean difference was > 20% only for ECOG status (21%), indicating good matching between the groups. There were no significant differences regarding demographic and clinical characteristics between the groups generated after PS matching. Comparison of matched groups is presented in Table 2.

**Table 2:** Demographic and clinical features after propensity score matching.

	Total n = 40	LR group n = 20	TACE group n = 20	Standardized mean difference (%)	*p* value
*Age (year)*	65.5 (29–80)	66.5 (42–80)	64.5 (29–77)	10	0.533
*Sex, n (%)*					
Male	24 (60)	12 (60)	12 (60)	0.1	1
Female	16 (40)	8 (40)	8 (40)		
*Cirrhosis, n (%)*					
Yes	25 (62.5)	12 (60)	13 (65)	10	1
No	15 (37.5)	8 (40)	7 (35)		
*Viral status, n (%)*					
HCV	20 (50)	10 (50)	10 (50)	0.1	1
HBV	14 (35)	7 (35)	7 (35)		
Negative	6 (15)	3 (15)	3 (15)		
*Child–Pugh score, n (%)*					
A	40 (100)	20 (100)	20 (100)	0.1	-
B	0	0	0		
*ECOG status, n (%)*					
0	39 (97.5)	20 (100)	19 (95)	21	1
1	1 (2.5)	0	1 (5)		
AFP (ng/mL)	347.5 (4–9898)	131.5 (4–5543)	481.5 (20–9898)	10	0.086
*Lesion number, n (%)*					
Solitary	36 (90)	18 (90)	18 (90)	0.1	1
Multiple	4 (10)	2 (10)	2 (10)		
*Distribution, n (%)*					
Unilobar	40 (100)	20 (100)	20 (100)	0.1	-
Bilobar	0	0	0		
*Major lesion size (cm), n (%)*					
10–14.9	24 (60)	12 (60)	12 (60)	0.1	1
15–19.9	12 (30)	6 (30)	6 (30)		
≥ 20	4 (10)	2 (10)	2 (10)		
*Albumin (g/L)*	40 (26–51)	41 (26–51)	39 (33–45)	6	0.854
*Total bilirubin (mmol/L)*	14 (5–39)	14 (6–36)	14 (5–39)	1	0.952
*ALT (IU/L)*	49 (6–206)	43 (16–166)	54 (6–206)	1	0.839
*γGT (IU/L)*	136.5 (16–470)	97 (16–470)	158 (29–272)	3	0.675
*PT*	12 (10–15)	12 (11–15)	12 (10–15)	8	0.677

Values are expressed as median (range) unless indicated otherwise.

*LR* liver resection, *TACE* transarterial chemoembolization, *HCV* hepatitis C virus, *HBV* hepatitis B virus, *ECOG* Eastern Cooperative Oncology Group, *AFP* alfa-feto protein, *ALT* alanine transaminase, *γGT* γ glutamil transferase, *PT* prothrombin time.

Before matching, the overall morbidity rate was higher in the LR group (64.7%) compared with the TACE group (37.1%), *p* = 0.012, while there was no difference after matching (LR, 60%; TACE, 30%), *p* = 0.055. The major morbidity, postintervention transfusion rate, in-hospital mortality and 30-days mortality were similar between the groups before and after matching. Perioperative outcome is shown in Table 3.

**Table 3:** Short-term treatment outcome before and after propensity score matching.

	Before PS matching				After PS matching			
	Total n = 103	LR group n = 68	TACE group n = 35	*p* value	Total n = 40	LR group n = 20	TACE group n = 20	*p* value
Overall morbidity	57 (55.3)	44 (64.7)	13 (37.1)	0.012	18 (45)	12 (60)	6 (30)	0.055
Major morbidity	18 (17.5)	14 (20.6)	4 (11.4)	0.287	5 (12.5)	2 (10)	3 (15)	1
Postinterventional transfusion	3 (2.9)	3 (4.4)	0	0.549	0	0	0	–
In-hospital mortality	3 (2.9)	3 (4.4)	0	0.549	0	0	0	–
30-days mortality	6 (5.8)	4 (5.9)	2 (5.7)	1	3 (7.5)	1 (5)	2 (10)	1

Values are expressed as absolute number (percentage).

*PS* propensity score, *LR* liver resection, *TACE* transarterial chemoembolization.

**Figure 2:**
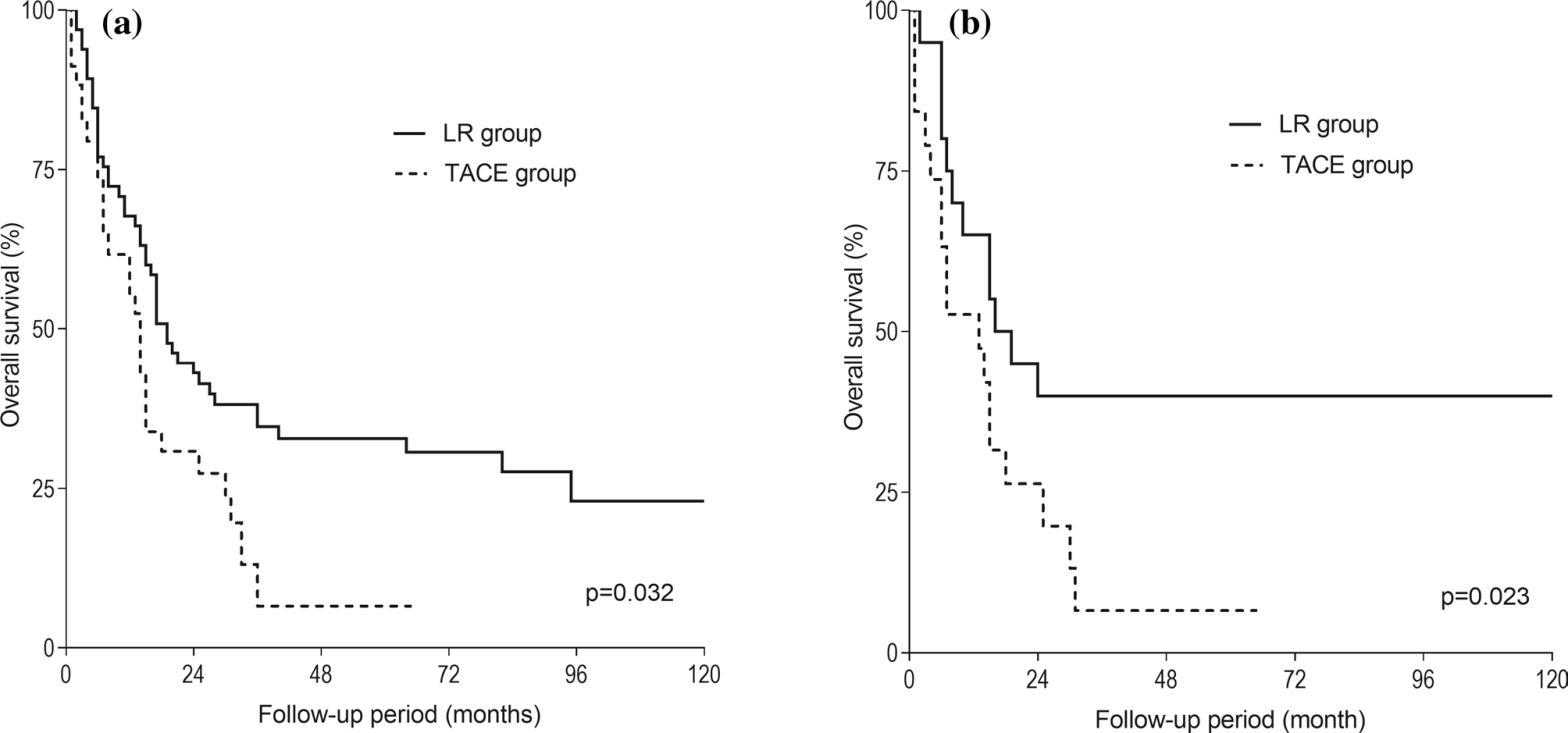
Overall survival curves of patients undergoing liver resection or transarterial chemoembolization before (**a**) and after (**b**) propensity match analysis. *LR* liver resection, *TACE* transarterial chemoembolization.

The median follow-up was 15 (range 0–155) months. In the LR group, 3-, 5- and 10-year survival was 36, 31 and 22%, respectively. In the TACE group, 3-, 5- and 10-year survival was 14, 7 and 0%, respectively. Overall survival was longer in the LR group than in the TACE group, *p* = 0.032 (Fig. 2a). After PS matching, the LR group showed longer 3-, 5-, and 10-year overall survival rates than the TACE group (40, 40 and 40% vs*.* 6, 6 and 0%, respectively), *p* = 0.023 (Fig. 2b).

Three potential predictors of poor long-term outcome of the matched cohort were identified by univariate analysis: multiple tumors (*p* = 0.044, hazard ratio (HR) = 3.221; 95%CI 1.034–10.030), total bilirubin (*p* = 0.022, HR = 1.061; 95%CI 1.008–1.116) and the TACE treatment (*p* = 0.032, HR = 2.242; 95%CI 1.073–4.684). Two of three factors were confirmed as independent predictors of long-term survival by multivariate analysis: total bilirubin (*p* = 0.029, HR = 1.061; 95%CI 1.006–1.119) and the TACE treatment (*p* = 0.038, HR = 2.203; 95%CI 1.044–4.649). Univariate and multivariate analysis are presented in Table 4.

**Table 4:** Univariate and multivariate analysis of prognostic factors associated with long-term survival in propensity score-matched patients (n = 40).

	Univariate analysis			Multivariate analysis		
	HR	95% CI	*p*	HR	95% CI	*p*
Age	1.003	0.973–1.034	0.852			
Sex (female)	0.979	0.471–2.034	0.955			
Cirrhosis	0.963	0.450–2.061	0.922			
ECOG	1.795	0.239–13.513	0.570			
AFP	1	1–1	0.658			
Tumor number (multiple)	3.221	1.034–10.030	0.044	2.522	0.804–7.913	0.113
Albumin	0.976	0.913–1.042	0.463			
Total bilirubin	1.061	1.008–1.116	0.022	1.061	1.006–1.119	0.029
ALT	0.998	0.990–1.005	0.545			
γGT	0.997	0.992–1.001	0.122			
PT	1.267	0.957–1.677	0.098			
Late study period	0.664	0.317–1.388	0.276			
Treatment (TACE)	2.242	1.073–4.684	0.032	2.203	1.044–4.649	0.038

*HR* hazard ratio, *CI* confidence interval, *ECOG* Eastern Cooperative Oncology Group, *AFP* alfa-feto protein, *ALT* alanine transaminase, *γGT* γ glutamil transferase, *PT* prothrombin time, *TACE* transarterial chemoembolization.

## Discussion

The presented study showed that in patients with huge HCC after PS matching, the LR group had longer 3-, 5-, and 10-year overall survival compared to the TACE group. The study confirmed that both treatment modalities were safe and feasible. However, LR has been shown as the more effective treatment modality, providing better long-term survival. Multivariate analysis indicated that total bilirubin and the TACE treatment are two independent predictors of long-term survival.

Available literature indicates that no randomized clinical trials comparing liver resection and TACE in patients with huge HCC have so far been done because a potentially effective treatment modality may be denied in one of the treatment arms^23^. To minimize the effects of confounding factors and to overcome a selection bias, PS matching was performed in the presented study. Comparison of baseline characteristics indicated important differences between the LR and the TACE groups regarding the tumor number and anatomical distribution through liver parenchyma. Furthermore, the greater frequency of multiple and bilobar tumors in the TACE group may directly affect overall survival as a potential confounding factor. PS analysis showed a poor balance between the two groups. Therefore, well-balanced treatment pairs were formed and, consecutively, a direct comparison of the safety and effectiveness of liver resection and TACE was performed.

Although the overall morbidity rate was higher in the LR group before PS matching, major morbidity (grade 3 or more) was similar before and after PS matching. This finding could be explained by strict adherence to the Clavien-Dindo classification system where minor complications were rigorously noted and classified. In the latest systematic review and meta-analysis by Wang and co-workers, 91 patients among 213 experienced postoperative morbidity, and mortality was 4.3%^24^. The results presented in this study are consistent with the results published in the literature. During the study period surgical skills and perioperative management were continuously improved. Importantly, the study was performed in an academic, high-volume center focused on selection of the optimal surgical candidate, which is essential for improving surgical outcomes.

Liver resection may improve long-term prognosis of patients with huge HCC compared with TACE. The study results indicated that 10-year survival can be achieved after liver resection for huge HCC. At the same time, 10-year survival was not achieved by any of the patients after TACE. Zhu and co-workers found better 5-year survival provided by hepatic resection than TACE for solitary huge HCC (38.7% vs. 20.8%, respectively)^21^. Min and co-workers observed longer 5-year overall survival before matching (39.8% vs. 9.7%, respectively), and longer 3-year survival after matching (40.2% vs. 18.5%, respectively)^22^. In the presented study, LR provided equal 5- and 10-year survival of 40% in the matched patients.

The presented study identified two independent predictors of poor overall survival among PS matched patients: total bilirubin and TACE treatment. In the study by Jin and co-workers a tumor larger than 8 cm and TACE treatment were associated with poor prognosis of HCC patients in BCLC stage A^25^.

The literature review identified three studies that investigated the efficacy of hepatic resection versus TACE for huge HCC^21,22,25^. PS matching analysis was applied in two of them; both studies concluded that LR is safe and more effective treatment than TACE for patients with solitary huge HCC^21,22^. However, the studies were performed in the Asia–Pacific region, where > 80% of HCC patients have chronic hepatitis B virus infection. Studies from the western world are missing. In Western countries, non-alcoholic steatohepatitis-related HCC is increasing, while viral-infection related cases are declining because of vaccination and effective anti-viral drugs^25^. The majority of HCC cases in Western countries have chronic hepatitis C virus infection as it was in the presented study (50% of patients after PS matching in both groups). The presented study was performed in a region characterized by a high percentage of HCC arising in non-cirrhotic livers, as has already been reported^26^.

Patients with HCC require a multidisciplinary approach regardless of the tumor burden because either liver resection or TACE applied as a mono-therapy provide limited long-term outcome. Moreover, the best treatment modality should be tailored according to the individual patient’s characteristics. In the era of precision medicine, molecular profiling rather than clinical staging play an important role in developing personalized treatment approach. Currently, the combined use of different treatment modalities seems to be more effective. Preoperative TACE in combination with portal vein embolization (PVE) is performed to aid the resectability of a huge HCC. The rationale of sequential TACE and PVE is that the absence of arterial flow prevents tumor enlargement while waiting for the hypertrophy as a result of PVE. Moreover, sequential approach is associated with the greater hypertrophy rate compared to PVE only^27^. Adjuvant TACE improves overall and disease-free survival targeting remaining arterial vessels to destroy cancer cells in a remnant liver, especially in patients with microvascular invasion^28^.

Preoperative selective internal radiotherapy with Yttrium-90 provided as bridging or downstaging modality was shown to be an effective treatment option prior to liver transplantation or resection. However, the embolization effect is less evident compared to TACE^29^. Systemic therapy i.e. chemotherapy and immunotherapy, is primarily intended for patients with advanced HCC. The goal of the targeted therapy in adjuvant settings is to eliminate microscopic disease or to prevent recurrent disease. Lately, there is a growing interest for immunotherapy in HCC patients because standard systemic therapy did not demonstrate meaningful efficacy against HCC. Different strategies combining immune checkpoint inhibitors with other systemic therapies or locoregional therapies are continuously being evolved to improve therapeutic response in patients with advanced HCC^30^. The role of systemic therapy for huge HCC needs to be clarified in the near future.

Recent advances in liver surgery are related to the use of minimally invasive techniques. Short- and long-term benefits of laparoscopic liver resection has been demonstrated over the last two decades^31^. The application of robotic technology offers a different approach from standard laparoscopy, tending to achieve a better control of the surgical field and to improve safety^32^. In robotic liver surgery, different skills are required including specific training in hepatobiliary surgery, knowledge on machine specification and underlying mechanisms. Future perspectives in the field of liver surgery are technological improvements such as real-time navigation and augmented reality in precision liver surgery.

This study has some limitations. The study methodology was created by a retrospective design. To overcome this drawback, prospectively maintained electronic databases for liver resection and TACE were used with records of all previously defined variables for every consecutive patient. Furthermore, PS matching analysis was performed to reduce the selection bias. Second, the presented study is a single-center analysis from a middle-income country. To increase sample size and to present entire cohort of HCC patients, a relatively long study period was used. During that period the management policies were gradually improved for both LR and TACE. Viral hepatitis treatment protocols were also changed in the meantime. Third, liver function parameters and general performance status may have confounding roles in the current analysis; therefore, further studies are needed to eliminate heterogeneity between the groups.

In conclusion, the study results indicate that in patients with huge HCC liver resection is associated with better long-term survival than with TACE. Therefore, surgical treatment should be considered as a first-line therapy whenever possible. Total bilirubin and TACE treatment are the two independent predictors of long-term survival.

## Methods

### Patients

From November 2001 until December 2018, 449 HCC patients were managed by a curative-intent LR or by TACE at the HPB unit of the University Clinic for Digestive Surgery, Clinical Center of Serbia, Belgrade. During the study period, 243 patients underwent 275 potentially curative liver resections. Among them, 32 patients were treated by second hepatectomy. From January 2008, when the TACE procedure was established, until 2018, 206 patients underwent 277 TACE interventions. The inclusion criteria for the study were: huge HCC (≥ 10 cm) treated either by a curative-intent liver resection or by palliative TACE. The exclusion criteria were: the initial treatment performed at another hospital, concomitant radiofrequency ablation and preoperative TACE followed by a curative-intent liver resection. Depending on the treatment modality, the patients were divided into either a liver resection group or a TACE group. All methods were carried out in accordance with relevant guidelines and regulations. The study protocol was approved by the Clinical Center of Serbia Institutional Review Board. Informed consent was obtained from all patients prior to the proposed type of treatment.

### Pretreatment investigations

HCC diagnosis was established according to EASL clinical practice guidelines^33^. Preoperative investigation included routine laboratory testing of blood count, biochemistry, coagulation, alpha-feto protein (AFP) and hepatitis viral B and C serology, transabdominal ultrasound, computed tomography and/or magnetic resonance imaging (MRI). Liver function was assessed by Child–Pugh score. Diagnosis of liver cirrhosis was established according to radiologic liver features. Upper endoscopy was conducted to evaluate the presence and the grade of esophageal varices. All patients underwent preoperative cardiology and anesthesiology assessment.

Demographic and clinical parameters were recorded: age, sex, cirrhosis, hepatitis viral status, Child–Pugh score, AFP, serum level of total bilirubin, alanine transaminase, γ glutamil transferase (γGT), prothrombin time (PT), albumin, lesion number, lobar distribution and major lesion size. Eastern Cooperative Oncology Group (ECOG) performance status was determined. The patients with liver cirrhosis were classified according to the BCLC staging.

### Surgical technique

Radiofrequency-assisted sequential “coagulate-cut” liver resection technique was used for parenchyma transection in the LR group^34^. Low central venous pressure and Pringle maneuver as inflow vascular control were not used routinely.

### Transarterial chemoembolization

With the patient under local anesthesia and using a trans-femoral approach, a 5 French (F) catheter is introduced into the superior mesenteric artery or common hepatic artery. A selective angiogram is then performed and the feeding arteries, tumor and vascular anatomy surrounding the tumor are identified. Then, a coaxial superselective microcatheter is inserted through the 5-F catheter as close to the tumor as possible. After the microcatheter is positioned in the target branch, an emulsion of 10–15 ml lipiodol (Andre Guerbet, Aulnay-sous-Bois, France) and 50–100 mg cisplatin is slowly injected through the catheter until blood flow is nearly stopped. The mixture of lipiodol and cisplatin was prepared by vigorously pumping the solutions 10–20 times between two syringes interconnected with a three-way stopcock. The doses of lipiodol and cisplatin were determined by the size and vascularity of the tumor. The course was repeated once every 1–2 months for 2–6 cycles.

Post-treatment morbidity was defined according to the Clavien-Dindo grading system^35^. A complication grade of 3 or more was considered as major morbidity. In-hospital mortality was defined as any death during the hospital stay, and 30-day mortality was any death within 30 days of the intervention.

### Treatment allocation and follow-up

The allocation of the specific treatment modality was based on tumor burden, future liver remnant (FLR) volume (both estimated on multi-detector computed tomography) and patient general health status. Liver resection was offered to patients in the following conditions: when negative resection margin was possible; feasibility of remaining at least two adjacent liver segments with independent portal and hepatic inflow, vein outflow and biliary drainage. Additionally, the minimal FLR was at least 40% of the total liver volume in patients with cirrhosis. In patients with no signs of cirrhosis, the minimal FLR volume was at least 30%. TACE was considered in patients with unresectable tumor, poor performance status or impaired liver function. All patients were presented to the multidisciplinary tumor board and decision for the specific treatment modality was made accordingly.

The follow-up after intervention included standard laboratory tests, AFP and transabdominal ultrasound every three months. CT or MRI was performed every six months. For the TACE group, CT scanning is performed one month after intervention to evaluate the effects of chemoembolization. After two years, follow-up visits were performed every six months.

The overall survival was calculated from the date of surgery in the liver resection group and from the date of TACE in the TACE group, until the day of death or until the last follow-up visit. Since TACE is a palliative treatment option disease-free survival was not estimated.

### Propensity score analysis

Propensity score (PS) matching was applied to reduce selection bias in this non-randomized study. A PS score was estimated for each patient separately using a logistic regression model. A model was constructed using covariates: age, sex, cirrhosis, viral status, Child score, ECOG status, AFP, albumin, total bilirubin, ALT, γGT, PT, lesion number, distribution and major lesion size. A one-to-one match using the nearest neighbor matching method without replacement was performed using a 0.2 caliper width. The balance between the groups was tested by standardized mean differences, where a difference smaller than or equal to 20% was considered a good balance indicator. Baseline characteristics, short-term outcomes and overall survival were compared before and after PS matching. PS matched patients were divided into the early and late study period group according to date of intervention (for LR group before and after January 1st 2010; for TACE group before and after January 1st 2013).

### Statistical analysis

Continuous variables were expressed as median (range). A Kolmogorov–Smirnov test was used to check the normality distribution of continuous data and then the independent samples T test or Mann–Whitney U test was applied. Categorical variables were expressed as absolute numbers (percentages) and compared using the χ^2^-test or Fisher’s exact test as appropriate. Overall survival was calculated using the Kaplan–Meier method. The difference between survival rates was assessed by the log-rank test. Univariate and multivariate analysis of prognostic factors for long-term survival in PS matched cohort were carried out using the Cox regression model. Significant prognostic factors identified by univariate analysis were incorporated into multivariate analysis and further assessed as independent prognostic factors.

Statistical analysis was carried out using SPSS version 23.0 (SPSS Inc., Chicago, IL, USA). Propensity score analysis and matching were performed with the psmatching program. All analyses were performed in R through the SPSS R-Plugin (SPSS R Essentials). *P* value ≤ 0.05 was considered statistically significant.

## References

[1] Bray F (2018). Global cancer statistics 2018: GLOBOCAN estimates of incidence and mortality worldwide for 36 cancers in 185 countries. CA Cancer J. Clin..

[2] Rawla P, Sunkara T, Muralidharan P, Raj JP (2018). Update in global trends and aetiology of hepatocellular carcinoma. Contemp. Oncol. (Pozn).

[3] Marcellin P, Kutala BK (2018). Liver diseases: a major, neglected global public health problem requiring urgent actions and large-scale screening. Liver Int..

[4] Forner A, Reig M, Bruix J (2018). Hepatocellular carcinoma. Lancet.

[5] management of hepatocellular carcinoma (2018). European Association for the Study of the Liver. Electronic address, e. e. e. & European Association for the Study of the, L. EASL clinical practice guidelines. J. Hepatol..

[6] Buonaguro L, Petrizzo A, Tagliamonte M, Tornesello ML, Buonaguro FM (2013). Challenges in cancer vaccine development for hepatocellular carcinoma. J. Hepatol..

[7] Global Burden of Disease Liver Cancer (2017). The burden of primary liver cancer and underlying etiologies from 1990 to 2015 at the global, regional, and national level: results from the global burden of disease study 2015. JAMA Oncol..

[8] Galun D (2015). Hepatocellular carcinoma: from clinical practice to evidence-based treatment protocols. World J. Hepatol..

[9] Lim C (2015). Hepatectomy for hepatocellular carcinoma larger than 10 cm: preoperative risk stratification to prevent futile surgery. HPB (Oxford).

[10] Mazzaferro V (1996). Liver transplantation for the treatment of small hepatocellular carcinomas in patients with cirrhosis. N. Engl. J. Med..

[11] Pavel MC, Fuster J (2018). Expansion of the hepatocellular carcinoma Milan criteria in liver transplantation: future directions. World J. Gastroenterol..

[12] Nault JC, Sutter O, Nahon P, Ganne-Carrie N, Seror O (2018). Percutaneous treatment of hepatocellular carcinoma: state of the art and innovations. J. Hepatol..

[13] Labgaa I, Demartines N, Melloul E (2019). Surgical resection versus transarterial chemoembolization for intermediate stage hepatocellular carcinoma (BCLC-B): an unsolved question. Hepatology.

[14] Peng ZW (2012). Hepatic resection versus transcatheter arterial chemoembolization for the treatment of hepatocellular carcinoma with portal vein tumor thrombus. Cancer.

[15] Chang YJ, Chung KP, Chang YJ, Chen LJ (2016). Long-term survival of patients undergoing liver resection for very large hepatocellular carcinomas. Br. J. Surg..

[16] Thelen A (2013). Liver resection for hepatocellular carcinoma in patients without cirrhosis. Br. J. Surg..

[17] Chung JW (2006). Transcatheter arterial chemoembolization of hepatocellular carcinoma: prevalence and causative factors of extrahepatic collateral arteries in 479 patients. Korean J. Radiol..

[18] Shrager B (2013). Resection of large hepatocellular carcinoma (>/=10 cm): a unique western perspective. J. Surg. Oncol..

[19] Hwang S (2015). Long-term outcome after resection of huge hepatocellular carcinoma >/= 10 cm: single-institution experience with 471 patients. World J. Surg..

[20] Miyayama S (2019). Outcomes of conventional transarterial chemoembolization for hepatocellular carcinoma >/=10 cm. Hepatol. Res..

[21] Zhu SL (2015). Efficacy of hepatic resection vs transarterial chemoembolization for solitary huge hepatocellular carcinoma. World J. Gastroenterol..

[22] Min YW (2014). Long-term survival after surgical resection for huge hepatocellular carcinoma: comparison with transarterial chemoembolization after propensity score matching. J. Gastroenterol. Hepatol..

[23] Resnik DB (2008). Randomized controlled trials in environmental health research: ethical issues. J. Environ. Health.

[24] Wang L, Liu Z, Liu X, Zeng Y, Liu J (2017). The hepatectomy efficacy of huge hepatocellular carcinoma and its risk factors: a meta analysis. Medicine (Baltimore).

[25] Jin YJ (2014). Surgery versus transarterial chemoembolization for solitary large hepatocellular carcinoma of BCLC stage A. J. Gastrointest. Surg..

[26] Galun D (2018). Preoperative neutrophil-to-lymphocyte ratio as a prognostic predictor after curative-intent surgery for hepatocellular carcinoma: experience from a developing country. Cancer Manag. Res..

[27] Tustumi F (2018). Preoperative strategies to improve resectability for hepatocellular carcinoma: a systematic review and meta-analysis. HPB (Oxford).

[28] Huo YR, Chan MV, Chan C (2020). Resection plus post-operative adjuvant transcatheter arterial chemoembolization (TACE) compared with resection alone for hepatocellular carcinoma: a systematic review and meta-analysis. Cardiovasc. Interv. Radiol..

[29] Eggert T, Greten TF (2017). Current standard and future perspectives in non-surgical therapy for hepatocellular carcinoma. Digestion.

[30] Xu W (2019). Immunotherapy for hepatocellular carcinoma: recent advances and future perspectives. Ther. Adv. Med. Oncol..

[31] Ciria R, Cherqui D, Geller DA, Briceno J, Wakabayashi G (2016). Comparative short-term benefits of laparoscopic liver resection: 9000 cases and climbing. Ann. Surg..

[32] Di Benedetto F, Petrowsky H, Magistri P, Halazun KJ (2020). Robotic liver resection: hurdles and beyond. Int. J. Surg..

[33] European Association for the Study of the Liver, European Organisation for Research and Treatment of Cancer (2012). EASL-EORTC clinical practice guidelines: management of hepatocellular carcinoma. J. Hepatol..

[34] Milicevic M (2007). A radiofrequency-assisted minimal blood loss liver parenchyma dissection technique. Dig. Surg..

[35] Dindo D, Demartines N, Clavien PA (2004). Classification of surgical complications: a new proposal with evaluation in a cohort of 6336 patients and results of a survey. Ann. Surg..

